# PATTERNS AND DETERMINANTS OF POLYSUBSTANCE USE AMONG RETIRED POPULATION IN A U.S. NATIONALLY REPRESENTATIVE SAMPLE

**DOI:** 10.1093/geroni/igz038.2552

**Published:** 2019-11-08

**Authors:** Tzung-Shiang Ou, Yi-Han Hu, Hsien-Chang Lin, Su-Wei Wong

**Affiliations:** 1 INDIANA UNIVERSITY, Bloomington, Indiana, United States; 2 Indiana University Bloomington, Bloomington, Indiana, United States

## Abstract

Polysubstance use in the U.S. has been a public health concern. The prevalence of substance use among middle-aged and older adults is predicted to increase by 50% by 2020. Previous studies revealed retirement could serve as a risk factor for substance use as this population was known to be more susceptible to mental health issues. However, patterns and determinants of polysubstance use among retired population remained understudied. The purpose of this study was to investigate the patterns and determinants of polysubstance use among retired adults aged 50 and older. This study extracted 3,019 retired participants from the 2017 National Survey on Drug Use and Health study. Polysubstance use was defined as the use of two or more substances, including alcohol, tobacco, marijuana, and painkiller misuse, in the past month. Weighted multinomial logistic regression model was conducted to examine the associations between utilization of mental health treatment and poly-use of substances. The findings suggested 52.0% of retired adults used at least one substance in the past month, where 17.0% used substances concurrently; 15% co-used alcohol, tobacco, and marijuana; 8.6% co-used tobacco and marijuana. Females were less likely to poly-use substances (RRR=0.37, p<.001) than males. Those who had received mental health treatment in the past year were more likely to co-use more than two substances in relative to substance non-users in the past month (RRR=1.71, p<.05). Retirement plan incorporating behavioral intervention and early detection of mental health issues are warranted to reduce polysubstance use among the retired population in the U.S.

